# Elevations of Extracellular Vesicles and Inflammatory Biomarkers in Closed Circuit SCUBA Divers

**DOI:** 10.3390/ijms24065969

**Published:** 2023-03-22

**Authors:** Awadhesh K. Arya, Costantino Balestra, Veena M. Bhopale, Laura J. Tuominen, Anne Räisänen-Sokolowski, Emmanuel Dugrenot, Erwan L’Her, Abid R. Bhat, Stephen R. Thom

**Affiliations:** 1 Department of Emergency Medicine, University of Maryland School of Medicine, Baltimore, MD 21201, USA; 2 Environmental, Occupational, Aging (Integrative) Physiology Laboratory, Haute Ecole Bruxelles-Brabant (HE2B), 1090 Brussels, Belgium; 3 DAN Europe Research Division, DAN Europe Foundation, 64026 Roseto degli Abruzzi, Italy; 4 Divers Alert Network, Durham, NC 27707, USA; 5 Laboratoire ORPHY, EA 4324, Université de Bretagne Occidentale UFR Science, 29238 Brest, France; 6 LaTIM INSERM UMR 1101, Université de Bretagne Occidentale UFR Science, 29238 Brest, France

**Keywords:** extracellular vesicles, exosomes, filamentous actin, decompression sickness, diving, hyperoxia, interleukin-1β, microglia, microparticles, nitric oxide synthase, plasma gelsolin

## Abstract

Blood-borne extracellular vesicles and inflammatory mediators were evaluated in divers using a closed circuit rebreathing apparatus and custom-mixed gases to diminish some diving risks. “Deep” divers (*n* = 8) dove once to mean (±SD) 102.5 ± 1.2 m of sea water (msw) for 167.3 ± 11.5 min. “Shallow” divers (*n* = 6) dove 3 times on day 1, and then repetitively over 7 days to 16.4 ± 3.7 msw, for 49.9 ± 11.9 min. There were statistically significant elevations of microparticles (MPs) in deep divers (day 1) and shallow divers at day 7 that expressed proteins specific to microglia, neutrophils, platelets, and endothelial cells, as well as thrombospondin (TSP)-1 and filamentous (F-) actin. Intra-MP IL-1β increased by 7.5-fold (*p* < 0.001) after day 1 and 41-fold (*p* = 0.003) at day 7. Intra-MP nitric oxide synthase-2 (NOS2) increased 17-fold (*p* < 0.001) after day 1 and 19-fold (*p* = 0.002) at day 7. Plasma gelsolin (pGSN) levels decreased by 73% (*p* < 0.001) in deep divers (day 1) and 37% in shallow divers by day 7. Plasma samples containing exosomes and other lipophilic particles increased from 186% to 490% among the divers but contained no IL-1β or NOS2. We conclude that diving triggers inflammatory events, even when controlling for hyperoxia, and many are not proportional to the depth of diving.

## 1. Introduction

The goal of this work was to improve the understanding of decompression sickness (DCS) pathophysiology. DCS is traditionally viewed as related to gas bubble formation from insoluble gas on decompression. However, the inconsistent presence of bubbles in human studies has prompted investigations that are focused instead on inflammatory pathways [1,2,3]. A body of work implicates a subset of extracellular vesicles (EVs), 0.1 to 1 µm microparticles (MPs), that are elevated in humans and rodent models exposed to high gas pressure and rise further after decompression [4,5,6,7,8,9,10,11,12,13,14,15]. MPs initiate a systemic inflammatory response related to neutrophil activation [13,16,17,18].

EVs are lipid bilayer-enclosed sub-cellular structures present in all bodily fluids that increase in association with inflammation [19]. EVs include exosomes (20–120 nm diameter particles generated by the endosomal pathway), 0.1–1 µm microparticles (MPs) generated by an outward budding of plasma membrane, and ~0.5–5 μm apoptotic bodies generated during cell self-destruction. Exosomes and MPs play roles in cell-to-cell communication due to their contents, which include nucleic acids, inflammatory mediators, and enzymes or organelles that generate free radicals [16,19,20,21]. 

This study evaluated inflammatory biomarkers in response to open water diving conducted by human subjects using closed circuit rebreathing (CCR) apparatus. This differs from typical self-contained underwater breathing apparatus (SCUBA) gear because exhaled gases are recycled after carbon dioxide removal and oxygen supplementation. Oxygen partial pressure is kept within narrow limits through the operation of intrinsic sensors. Breathing air at a high pressure involves exposure to elevated partial pressures of oxygen, nitrogen, and other respired gases. CCR is often used with custom-mixed gases rather than air to diminish the risks of oxygen toxicity and nitrogen narcosis. Thus, our rationale was to evaluate EVs production and other manifestations of inflammation in a cadre of open-water divers, where some of the confounding variables posed by breathing air were diminished due to CCR utilization. 

High pressures of nitrogen and noble gases such as helium and argon activate leukocytes via an oxidative stress process that triggers MPs production [22]. Exposure to high gas pressure and subsequent decompression pose a dual insult. Studies with human volunteers have demonstrated that while under pressure and before decompression, there are elevations in MPs number and those expressing filamentous (F-) actin on the membrane surface [5,23]. Similar responses occur in a murine DCS model, and when these MPs are purified and injected into naïve mice, they cause the same spectrum of injuries as seen in decompressed mice [13,15,23]. The second insult occurs because some MPs generated due to high gas pressure are enriched with inflammatory nitric oxide synthase (NOS)2. Enzyme activity within the MPs is responsible for generating a gas phase which provides a nucleation site for inert gas uptake on decompression. This causes MPs to enlarge, and they then cause vascular damage and neutrophil activation that can be abrogated by reversing particle enlargement [16,17,18].

The mechanism for the dual risk of MPs is related to neutrophil responses. As mentioned, some MPs formed in response to high gas pressures have F-actin on the surface. These particles will exacerbate MPs production because they trigger neutrophil auto-activation [24]. The rigidity caused by the MPs F-actin shell allows phosphatidylserine that is ubiquitously present in the MPs membrane to be recognized by a complex of receptors, including CD36, Toll-like receptor (TLR)4, and the receptor for advanced glycation end-products (RAGE). These proteins are linked to a scaffold protein called NOS1 adaptor protein. When the receptors bind to a MP, an increase in membrane colocalization occurs and NOS2 binds to the complex [24,25]. NOS2 facilitates S-nitrosylation of Src kinases and the cytoskeleton, resulting in formation of the nucleotide-binding domain leucine rich repeat (NOD)-like receptor, pyrin containing 3 (NLRP3) inflammasome. The NLRP3 inflammasome is responsible for producing mature interleukin (IL)-1β [15,22,25]. Inflammasome assembly correlates with MP production, and the MPs that contain high amounts of IL-1β are a factor in diffuse vascular damage in a murine DCS model [15,26]. It is unclear whether the MPs expressing F-actin are the same as those containing NOS2. 

## 2. Results

### 2.1. Study Subjects and Protocol

**Figure 1:**
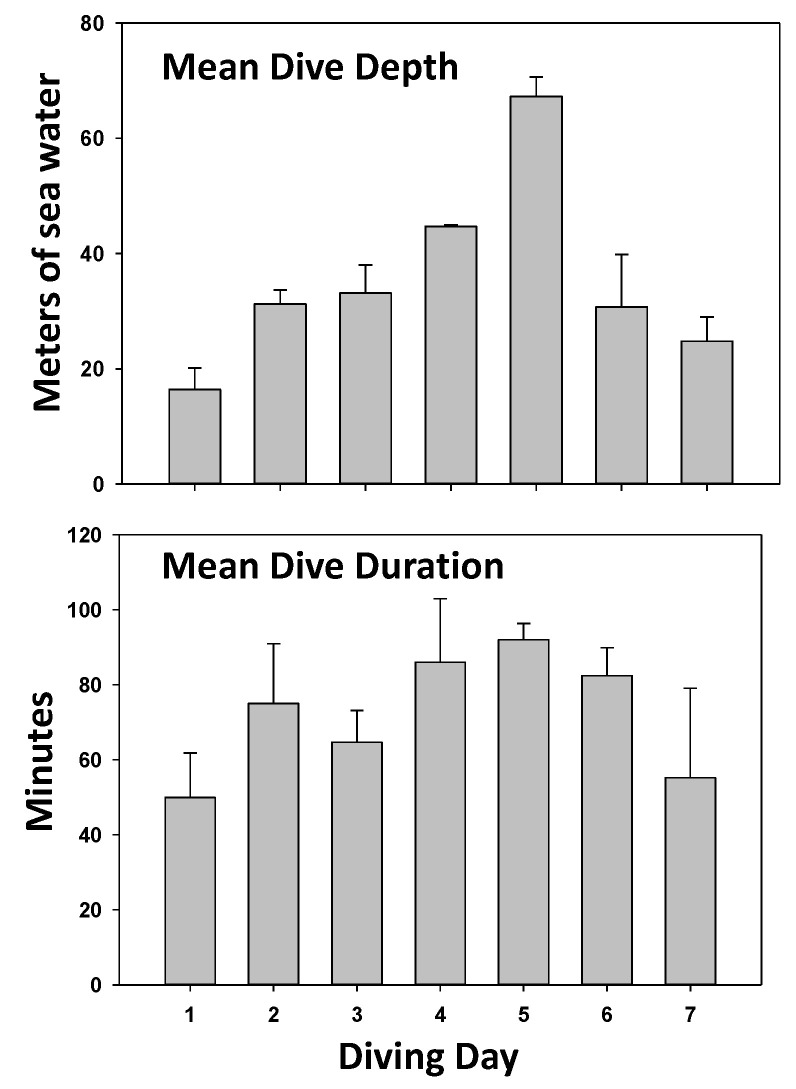
Diving activity in the shallow diver group. Data show diving depth and duration (mean ± SD) for the 6 individuals in the shallow diver group. On day 1, all performed 3 dives; on days 2 and 3, all performed 2 dives; on day 4, only 5 of 6 each performed one dive; on day 5, all 6 performed 1 dive; on day 6, three divers each performed 2 dives; and on day 7, four divers performed 2 dives.

This investigation involved two groups of CCR divers. One group of 8 deep divers performed a single dive to a mean depth of 102.5 ± 1.2 (SD) meters of sea water (msw) for 167.3 ± 11.5 min. The diluent gas in the CCR apparatus was a mixture of 10% oxygen, 20% nitrogen, and 70% helium (used to diminish the risk of nitrogen narcosis). A second group of 6 divers performed a series of 9 to 12 dives at lesser depths over a span of 7 days (termed shallow group) using a CCR apparatus with 18% nitrogen, 50% helium, and the balanced oxygen. All divers in this group did three dives on day one, to allow for comparisons with the deep divers, and repetitive dives for the subsequent week. The daily activity in this group is illustrated in Figure 1. The mean diving depth for the shallow group was 16.4 ± 3.7 msw, with a mean time of 49.9 ± 11.9 min. For both groups of divers, the CCR apparatus was set to maintain constant oxygen partial pressure at 120 kPa, and a built-in safety threshold kept the level greater than 140 kPa during the shallow return phase of dives (to accelerate inert gas removal). There were no diving mishaps and none of the 14 divers sustained signs or symptoms of DCS.

### 2.2. Blood-Borne EVs Elicited by Diving

Blood counts are shown for MPs in Table 1. MPs were identified based on the size (0.1–1 µm) and surface expression of annexin V (which binds to phosphatidylserine, Table 1). In addition to the total number of particles, the expression of surface proteins on MPs originated from neutrophils (CD66b), platelets (CD41), endothelial cells (CD146), and microglia (TMEM119) were analyzed. To gain further insight into MPs, surface expression of thrombospondin-1 (TSP) and staining with phalloidin were also assessed. Phalloidin binding is an index of F-actin expression, and recent work in the murine model has indicated an inflammatory role for TSP-expressing MPs [27]. Both dive profiles caused significant elevations in total MPs, whereas only the deep divers also showed significant elevations in the various MPs subgroups, expressing cell-specific proteins after the first day of diving. However, after the 7-day course of repetitive diving, the shallow group divers exhibited significant elevations in subgroups versus pre-dive values (Table 1). 

Exosomes were enumerated (Table 2), as described more extensively in Methods, as particles with diameters between 20 and 120 nm that were stained with lipophilic PKH67 dye, and subsets were analyzed for the expression of several tetraspanin proteins. The analysis involves an initial evaluation based on particle size, and additional steps are necessary to separate bilayer-enclosed particles from random debris. We used the PKH67 dye as our prime stain, which has been used by others [28]. In preliminary studies, we found PKH stained 52.1 ± 11.4% (*n* = 9) of all 20–120 nm diameter structures, whereas an alternative lipid stain, Laurdan, only detected 22.2 ± 6.9% in pre-diving samples. Laurdan detects changes in lipid phase properties through its sensitivity to bilayer polarity. After diving, PKH stained 51.7 ± 19.2% (NS, versus pre-dive), whereas Laurdan stained 75.5 ± 5.2% (*p* < 0.001 versus pre-dive, *t*-test) of all 20 to 120 nm diameter particles. The Laurdan emission spectrum maximum is centered at 490 nm in a disordered (gel) phase and 430 nm in a packed (liquid crystalline phase) [29]. When selecting 20 to 120 nm PKH-staining particles, prior to diving 41.3 ± 26.0% also stain with Laurdan, but after diving all stained positive (the bandpass filter allowed passage of 457 ± 45 nm light). 

Exosome membranes are rich in tetraspanins, endosomal proteins that organize membrane microdomains. Several were evaluated in the shallow diver group (inadequate sample volumes precluded assays from the deep divers) among PKH-positive particles between 20 and 120 nm in diameter. CD63 expression pre-dive occurred on 74.7 ± 2.4%, and 94.7 ± 0.6% after day 1, and 94.6 ± 3.5 % after day 7 (*p* < 0.001 versus pre-diving, RM-ANOVA). CD81 expression pre-dive occurred on 67.7 ± 2.4%, 93.3 ± 0.4% after day 1, and 94.5 ± 4.1% after day 7 (*p* < 0.001 versus pre-diving, RM-ANOVA). 

### 2.3. Neutrophil Activation Elicited by Diving

The activation of neutrophils, which express the CD66b protein, was assessed by flow cytometry as the surface expression of the CD18 protein component of the β_2_ integrin, or myeloperoxidase (MPO) above background on CD66b-positive cells. Neither diver group exhibited activation after day 1. Only after the 7th day of diving was there a statistically significant increase in cells expressing MPO in the shallow diver group (Table 3).

### 2.4. IL-1β in MPs Increased by Diving

IL-1β secretion requires unconventional pathways, involving packaging into either MPs or exosomes, to be liberated to the extracellular milieu [30]. To assess cargo differences in MPs versus exosomes, the plasma preparation used for enumerating EVs were centrifuged at 21,000× *g* for 30 min. Afterwards, 67.6 ± 17.8 (*n* = 13) % of all MPs and only 15.5 ± 5.2 (*n* = 13) % of the exosomes/lipophilic particles were found in the pellet. There was no detectable IL-1β in 21,000× *g* supernatants, indicating the absence of this cytokine in exosomes. Contents in pellets are shown in Table 4. There was a statistically significant difference between the pre-dive IL-1β levels in the deep versus the shallow divers, which suggests some pre-diving differences between groups. Within each group, there were significant differences in values on day 1 diving in the deep divers, and at day 7 in the shallow diver group (Table 4). 

### 2.5. NOS2 in MPs Increased by Diving

Animal studies suggest there is a role for NOS2 generating a gas phase in MPs [16]. Others have reported NOS activity and presence of NOS2 in exosomes [31,32]. There was no detectable NOS2 in the 21,000× *g* supernatant preparation (exosomes exosomes/lipophilic particles and few MPs), but there were significant elevations versus pre-dive values in the pellets, as shown in Table 5. 

### 2.6. Plasma Gelsolin (pGSN) Decreased by Diving

Plasma gelsolin is a highly conserved, cytoplasmic actin-binding protein that has been reported to decrease in post-diving samples in a murine DCS model and in human subjects exposed to pressure in a hyperbaric chamber [23]. The open-water diver data shown in Table 6 demonstrate significant reductions in pGSN post-dive in the deep divers at day 1, and among the shallow divers at day 7. 

## 3. Discussion

This study demonstrates that MPs elevations and neutrophil activation in CCR divers mirror changes previously reported in air-breathing divers, where oxygen partial pressures were variable and often higher [4,5,7,8,9,11,12,14]. Changes in MPs subgroups expressing different cell-specific proteins and concurrent changes in IL-1β, NOS2, and pGSN provide additional insight. Based on the mechanisms of MPs production by high pressure gases, one would anticipate that exposure to higher pressures will trigger more MPs to be formed than in lower gas pressures, as was observed. However, helium triggers less oxidative stress than an equal pressure of nitrogen, so one cannot directly compare responses between the diver groups [22]. It is notable that elevations in MPs expressing microglial-specific TMEM were found after the single ~100 msw dive, whereas a similar elevation in shallow divers was only observed after 7 days of repetitive diving. We recently reported that TMEM-expressing MPs that also expressed TSP generated in the brain are released via the brain glymphatic system to the systemic circulation, where they activate neutrophils to generate a second array of MPs, some of which express F-actin (29). This cascade of MPs responses could explain differences between the diver groups. MPs-bearing proteins specific to neutrophils (CD66b), endothelial cells (CD146), and platelets (CD41a) support activation of these cells from diving. We cannot rule out a role for platelet-derived TSP in the elevation of MPs expressing this protein in the deep diver group. 

We also report, for the first time, exosome/lipophilic particle elevations in response to diving. We assessed these particles based on their 20 to 120 nm diameter and staining with the lipophilic PKH67 dye. There is a complex interplay between exosomes, the NLRP3 inflammasome, and IL-1β. Inflammatory stimuli can enhance exosome formation and exosomes can either enhance or inhibit inflammasome formation and IL-1β production [33]. Mechanism (s) for exosome formation with diving will require further investigation, but could be linked to MPs, given their IL-1β cargo. We found that there were higher percentages of sub-micron particles expressing CD63 and CD81 post-diving, supporting the presence of exosomes versus merely non-specific lipophilic particles. We also utilized Laurdan staining in the protocol. The changes in CD63 and 81 and Laurdan staining are indicative of new exosome populations with differing protein and lipid content. The higher percentage of particles detected by Laurdan in the post-dive samples is likely due to the emission spectrum of the dye (490 nm in a disordered lipid [gel] phase versus 430 nm in an ordered [liquid crystalline] phase), and because the detection channel bandpass filter in the flow cytometer was 475 ± 45 nm. This is not the same as providing direct information about lipid packing domains (so-called lipid rafts) which requires more precise dual wavelength measurements to assess the generalized polarization [34].

Plasma gelsolin (pGSN) blood levels fall in numerous acute and chronic inflammatory states. Among studies, the magnitude of pGSN reduction parallels the extent of tissue damage, and depletion precedes and predicts adverse clinical outcomes [35,36,37,38,39,40,41]. In the present study, we observed significantly lower pGSN concentrations in post-dive plasma. Prior work suggests that the decrease occurs due to pGSN consumption from binding and lysis of F-actin-expressing MPs. In the murine DCS model, and numerous animal studies of infection, injury, and inflammation, pGSN supplementation can abrogate organ damage [23,42]. Our study is the first to show reductions of pGSN in open-water divers.

Elevations of IL-1β following high gas pressure exposure have been described in the murine model and in humans after simulated diving [5,15,26]. Here, we show IL-1β elevations in CCR divers, and that it is present in MPs but not in exosomes. This is consistent with NLRP3 inflammasome assembly occurring concurrently with MPs formation in response to high gas pressure exposures. We also demonstrate NOS2 presence in human MPs and an increased content post-diving. When neutrophils are stimulated by binding MPs, synthesis of many proteins associated with NLRP3 inflammasome formation are increased, including NOS2 [24,25]. This has important implications for diving physiology because NOS2 is a high-output Ca^++^-independent NOS isoform. After expressional induction, it continuously produces nitric oxide until the enzyme is degraded. This alone (separate from other changes post-diving) can impair normal vascular tone and endothelial integrity [31,32]. Moreover, murine studies indicate that MPs NOS2 is responsible for generating a gas phase of NO_2_ (from oxidized nitric oxide) within MPs [16]. As these MPs enlarge on decompression, the data suggest that enzyme activity provides a nucleation site for bubble formation. The findings in this project–that MPs are more numerous and possess higher NOS2 concentrations post-dive, somewhat proportional to dive depth–imply greater potential for bubble nucleation and greater risk for bubble-induced vascular damage. It should be noted that, given the micro-dimensions of MPs, bubbles related to MPs (that would be expected to lyse with sufficient growth) would still be below the detection limits of current ultrasound technology.

This project reconfirms the pro-inflammatory effects of diving in humans. It also, for the first time, documents elevations in exosomes/lipophilic particles, although their pathophysiological role in diving remains unclear. It should be emphasized that divers in this study exhibited no adverse health effects. Oxidative stress and inflammatory responses are not necessarily manifestations of toxicity and organ damage. However, all changes seen in these research subjects are directly linked to tissue damage in the murine DCS model. Clearly, therefore, additional events are necessary for symptom development and overt DCS. An attractive hypothesis is that some individuals may exhibit lower pGSN at baseline and/or more exuberant NOS2 production and activity, such that nucleation-site-carrying MPs generate more intravascular bubbles or carry more IL-1β in response to diving. These issues are currently under investigation. 

## 4. Materials and Methods

### 4.1. Experimental Protocol

All subjects gave their informed consent for inclusion before they participated in the study. The study was conducted in accordance with the Declaration of Helsinki, and the protocol was approved by the Bio-Ethical Committee for Research and Higher Education, Brussels (Nº B200-2020-088). Analyses of deidentified blood samples were approved by the University of Maryland Institutional Review Board (Nº HP-00059996). After written, informed consent, 13 male, healthy, non-smoking divers (Minimum certification “Autonomous Divers” according to European norm EN 14153-2 or ISO 24801-2 with at least 50 logged dives) volunteered for this study. None of them had a history of previous cardiac abnormalities or were under any cardio- or vaso-active medication. They were selected from a large population of divers in order to have a homogenous sample: aged 44.7 ± 12.4 years old (mean ± SD); height 173 cm ± 6.6; weight 75.2 ± 13.7 kg. 

### 4.2. Reagents

Chemicals were purchased from Sigma–Aldrich (St. Louis, MO, USA) unless otherwise noted. Annexin-binding buffer and the following agents were purchased from BD Pharmingen (San Jose, CA, USA): fluorescein isothiocyanate (FITC) conjugated Annexin V (cat # 556419), R-PE conjugated anti-human CD18 (cat # 555924), and PerCP/Cy5.5 conjugated anti-human CD41a (cat # 340931). APC-conjugated anti-human CD146 (cat # 340931) was purchased from BioScience (San Diego, CA, USA), AlexaFluor488-conjugated anti-human TMEM119 (cat # FAB103131G) was from R & D Systems (Minneapolis, MN, USA), anti-thrombospondin (TSP)-1 (cat # sc-393504) was from Santa Cruz Biotechnology (Dallas, TX, USA), and FITC-conjugated anti-human myeloperoxidase (MPO, cat # HM1051PE-100) was from Hycult Biotech (Plymouth Meeting, PA, USA). Antibodies purchased from Biolegend (San Diego, CA, USA) included: AlexaFluor647-conjugated anti-human CD63 (cat # 353016), PercpCy5.5-conjugated anti-human CD81 (cat # 349520), and BV421-conjugated anti-human CD66b (cat # 347201).

### 4.3. Blood Sampling and Laboratory Procedure

Blood samples were obtained before and 120 min after the last dive on the first day from both diver groups (deep and shallow), and after the last dive on the 7th day for the shallow group. Blood (~5 mL) was drawn into Cyto-Chex BCT test tubes that contained a proprietary preservative (Streck, Inc., Omaha, NE, USA). Samples were sent by express mail to the University of Maryland (Dr. Thom) laboratory, where all analyses were performed following published techniques described in previous publications [25,43]. In brief, total MPs and subtypes were assayed in an 8-color, triple-laser MACSQuant (Version 2.13.3, Miltenyi Biotec Corp., Auburn, CA, USA) flow cytometer with the manufacturers’ acquisition software using standard methods, including a “fluorescence minus one control test” [44]. This analysis provides a way to define the boundary between positive and negative particles in an unbiased manner by defining the maximum fluorescence expected for a given subset after outlining the area in a two-dimensional scatter diagram when a fluorophore-tagged antibody is omitted from the stain set. The analysis allows for a simple decision as to where to place the upper boundary for non-staining particles in a fluorescence channel. All supplies, reagents, and manufacturer sources have been described in previous publications [8,9,11,12]. 

The blood was centrifuged for 5 min at 1500× *g*, the supernatant was made to 12.5 mmol/L EDTA to impede MP aggregation, and then centrifuged at 15,000× *g* for 30 min. Aliquots of the 15,000× *g* supernatant were stained with antibodies for MP analysis by flow cytometry, and a portion was used for exosome analysis. Plasma stored at −80 °C after a 15,000× *g* centrifugation step preceding MP analysis was used for IL-1β, NOS2, and pGSN assays.

### 4.4. Neutrophil Activation Analysis 

Whole fixed blood from the Cyto-Chex tubes (100 µL) was stained for 30 min at room temperature in the dark with optimized concentrations of antibodies as listed above. After staining, 2 mL phosphate buffered saline (PBS) was added to dilute each sample tube prior to analysis, with the cytometer acquisition set to use anti-human CD66b as the fluorescence trigger to recognize neutrophils.

### 4.5. IL-1β and NOS2 Measurements 

Human-specific ELISA Kits (eBioscience, San Diego, CA, USA) that detect pro- and mature forms of IL-1β or NOS2 were used following the manufacturer’s instructions. Measurements were made using plasma supernatant after blood was centrifuged at 15,000× *g*, as described for flow cytometry studies, and also in supernatant and pellet fractions separated by a second centrifugation at 21,000× *g* for 30 min. The MPs in pellets were placed in a 0.3 mL lysis buffer, the protein content of the sample was measured, diluted to 5 mg/mL, and 20 µg protein was used for analysis. 

### 4.6. Gelsolin Assay

A human-specific commercial pGSN ELISA kit (LSBio, Inc. Seattle, WA, USA) was used following the manufacturer’s instructions. Serial dilutions in PBS were prepared using the supernatant after 15,000× *g* centrifugation of plasma, as described above, and analyzed concurrent with a range of known pGSN standards.

### 4.7. Exosome/Lipophilic Particles Assay

Using 15,000 g supernatants from plasma as described above, 5 µL samples were diluted in 100 µL PBS and incubated with dyes [2.5 µmol PKH67 (Sigma Cat#SIG-MINI67), 2.5 µmol Laurdan (1-[6-(Dimethylamino)-2-naphthalenyl]-1-dodecanone, Tocris Biotech Cat#7275)], and antibodies for 30 min prior to analysis using an ImageStream^®^X Mk II: Imaging Flow cytometer. FluoSphere™ carboxylate-modified microspheres (Thermofisher, 20 and 100 nm in diameter) were used to provide size bracketing, and initial standardization of methods was conducted with 120 nm (range 80–140 nm) synthetic lipid vesicles from Cellarcus Biosciences (San Diego, CA, USA) that were made with a lipid composition comparable to mammalian cells. For initialization, the instrument bright field and lasers were set to maximum power, side scatter (SSC) set to 70 mW, and the 60× imager magnification set to high gain. Channel 1 was used for bright field and Ch.6 for SSC. After setting the compensation matrix with bright field off and all channels enabled, single-color compensations were set for each color, with gates set to detect particles between 20 and 100 nm in diameter.

### 4.8. Statistical Analysis

Results are expressed as the mean ± SD. Data were analyzed using SigmaStat (Version 12.5, Jandel Scientific, San Jose, CA, USA). The normality of the data was assessed with the Shapiro–Wilk tests, and if passed, data was analyzed with a Student’s *t*-test between groups and repeated measures analysis of variance (RM-ANOVA) with the post-hoc Tukey test, where appropriate. A small number of data sets for the deep diver group failed the normality test, and comparisons within the group were performed by means of the non-parametric Mann–Whitney test. For all studies, we deemed a result to be statistically significant if *p* < 0.05. 

## Figures and Tables

**Table 1:** Microparticles in blood. Flow cytometry was used to evaluate MPs. Total MPs/µL plasma is shown in the first column, and other columns show the percent of each that expressed proteins specific to different cells, including neutrophils (CD66b), endothelial cells (CD146), platelets (CD41a), and microglia (transmembrane protein 119, TMEM). As discussed in the text, proteins expressing TSP-1 and F-actin, evaluated as those binding phalloidin, were also assessed. Rows show values for divers in the deep group prior to their dive and after the ~100 m dive. The next three rows show results for divers in the shallow group prior to their first dive, after the first day of 3 dives, and after the last of 7 days diving. Data are mean ± SD (*n* = number of diver’s samples; + *p* < 0.001, *t*-test; * *p* < 0.001 vs. control, RM-ANOVA). Bold numbers are to indicate statistically significant values.

	MPs/µL	% CD66b	%CD146	%TSP	%CD41	%TMEM	%Phalloidin
Deep-Pre (8)	900 ± 68	11.0 ± 0.5	25.1 ± 0.5	12.7 ± 0.4	3.5 ± 0.4	31.3 ± 0.9	17.3 ± 0.2
Deep-Post (8)	**1089 ± 68 +**	**14.3 ± 2.0 +**	**28.4 ± 1.8 +**	**15.9 ± 2.3 +**	**5.7 ± 1.6 +**	**34.4 ± 2.1 +**	**20.0 ± 1.9 +**
Shallow-pre (6)	799 ± 68	11.4 ± 2.6	23.0 ± 2.5	13.6 ± 2.5	3.1 ± 1.1	29.2 ± 2.0	18.4 ± 1.5
Shallow- 1 day post (6)	947 ± 31 *	12.8 ± 1.5	23.6 ± 1.5	14.5 ± 1.4	4.8 ± 1.9	29.9 ± 0.8	19.4 ± 1.3
Shallow- 7 day post (6)	1016 ± 48 *	18.7 ± 1.5 *	27.4 ± 1.5 *	16.2 ± 1.8	7.1 ± 1.8 *	33.2 ± 1.7 *	20.7 ± 1.5

**Table 2:** Exosomes/lipophilic particles in blood. Imaging flow cytometry was used to evaluate exosomes and lipophilic particles as total number/µL (mean ± SD, *n* = number of samples) pre- and post-deep and shallow dives. Note that due to insufficient plasma volume, analysis could only be conducted on 4 of the 8 participants in the deep diver group (+ *p* < 0.001, *t*-test; * *p* < 0.001 vs. control, RM-ANOVA). Bold numbers indicate statistically significant values.

	Pre-Dive (#/µL)	Post-Day 1 (#/µL)	Post Day 7 (#/µL)
Deep divers (*n* = 4)	9.3 ± 0.9 × 10^7^	**16.5 ± 5.2** × **10^7^ +**	
Shallow divers (*n* = 6)	6.1 ± 2.6 × 10^7^	14.7 ± 3.6 × 10^7^ *	17.3 ± 3.8 × 10^7^ *

**Table 3:** Activation of neutrophils from all divers in both groups. Data are the mean ± SD (*n* = sample number) % of neutrophils (identified in the flow cytometer based on CD66b expression) expressing myeloperoxidase (MPO) and CD18 above a threshold value as an index of cell activation. Pre- and post-day 1 are values from the deep and shallow groups, and those in the third column reflect values from the shallow group, and the statistical analysis compared against the values only takes this group into account (pre- and post-day 1 as repeated measures ANOVA. (* *p* < 0.001 vs. pre-dive, RM-ANOVA).

	Pre-Dive	Post-Day 1	Post Day 7
% MPO	8.7 ± 4.0 (13)	8.7 ± 6.1 (13)	10.7 ± 5.4 (6) *
% CD18	2.1 ± 1.9 (13)	4.5 ± 3.4 (13)	3.7 ± 4.7 (6)

**Table 4:** Intra-MPs IL-1β as pg/million MPs. Data are the mean ± SD (*n* = sample number) pre- and post-diving for both diver groups (+ *p* < 0.02, *t*-test; * *p* < 0.001 RM-ANOVA; ^1^ *p* < 0.01 *t*-test within columns).

	Pre-Dive	Post-Day 1	Post-Day 7
Deep divers (8)	10.0 ± 9.5 ^1^	65.6 ± 23.1 + ^1^	
Shallow divers (6)	3.1 ± 1.5	21.6 ± 16.5	104.9 ± 14.0 *

**Table 5:** Intra-MPs NOS2 as pg/million MPs. Data are the mean ±SD (*n* = sample number) pre- and post-diving for both diver groups (+ *p* ≤ 0.02, *t*-test; * *p* < 0.001 RM-ANOVA.

	Pre-Dive	Post-Day 1	Post-Day 7
Deep divers (8)	0.03 ± 0.06	0.17 ± 0.12 +	
Shallow divers (6)	0.04 ± 0.06	0.16 ± 0.11 *	0.17 ± 0.06 *

**Table 6:** Plasma gelsolin (µg/mL). Data are the mean ± SD (*n* = sample number) pre- and post-diving for both diver groups (+ *p* < 0.001, *t*-test; * *p* < 0.001 RM-ANOVA; There is not a statistically significant difference in pre-dive values between the two groups).

	Pre-Dive (µg/mL)	Post-Day 1	Post-Day 7
Deep divers (8)	121.3 ± 33.1	34.8 ± 20.4 +	
Shallow divers (6)	168.5 ± 21.5	151.9 ± 13.7	106.7 ± 30.9 *

## Data Availability

Data are available at request from the authors.

## Abbreviations

CCR	Closed circuit rebreather
DCS	Decompression sickness
EVs	Extracellular vesicles
IL-1β	Interleukin-1β
MPO	Myeloperoxidase
MPs	Blood Borne Microparticles
NLRP3	Nucleotide-binding domain leucine rich repeat (NOD)-like receptor, pyrin containing 3 inflammasome
NOS2	Nitric oxide synthase-2
pGSN	Plasma gelsolin
PO_2_	Oxygen Partial Pressure
RAGE	Receptor for advanced glycation end products
TMEM	Transmembrane protein 119
TLR4	Toll-like receptor 4
TSP	Thrombospondin 1

## References

[1] Francis T.J., Pearson R.R., Robertson A.G., Hodgson M., Dutka A.J., Flynn E.T. (1988). Central nervous system decompression sickness: Latency of 1070 human cases. Undersea Hyperb. Med..

[2] Bigley N.J., Perymon H., Bowman G.C., Hull B.E., Stills H.F., Henderson R.A. (2008). Inflammatory cytokines and cell adhesion molecules in a rat model of decompression sickness. J. Interferon Cytokine Res..

[3] Martin J.D., Thom S.R. (2002). Vascular leukocyte sequestration in decompression sickness and prophylactic hyperbaric oxygen therapy in rats. Aviat. Space Environ. Med..

[4] Barak O.F., Janjic N., Drvis I., Mijacika T., Mudnic I., Coombs G.B., Thom S.R., Madic D., Dujic Z. (2020). Vascular dysfunction following breath-hold diving. Can. J. Physiol. Pharmacol..

[5] Brett K.D., Nugent N.Z., Fraser N.K., Bhopale V.M., Yang M., Thom S.R. (2019). Microparticle and interleukin-1B production with human simulated compressed air diving. Sci. Rep..

[6] Vince R.V., McNaughton L.R., Taylor L., Midgley A.W., Laden G., Madden L.A. (2009). Release of VCAM-1 associated endothelial microparticles following simulated SCUBA dives. Eur. J. Appl. Physiol..

[7] Madden L.A., Chrismas B.C., Mellor D., Vince R.V., Midgley A.W., McNaughton L.R., Atkins S.L., Laden G. (2010). Endothelial function and stress response after simulated dives to 18 msw breathing air or oxygen. Aviat. Space Environ. Med..

[8] Thom S.R., Milovanova T.N., Bogush M., Bhopale V.M., Yang M., Bushmann K., Pollock N.W., Ljubkovic M., Denoble P., Dujic Z. (2012). Microparticle production, neutrophil activation and intravascular bubbles following open-water SCUBA diving. J. Appl. Physiol..

[9] Thom S.R., Milovanova T.N., Bogush M., Yang M., Bhopale V.M., Pollock N.W., Ljubkovic M., Denoble P., Madden D., Lozo M. (2013). Bubbles, microparticles and neutrophil activation: Changes with exercise level and breathing gas during open-water SCUBA diving. J. Appl. Physiol..

[10] Pontier J.M., Gempp E., Ignatescu M. (2012). Blood platelet-derived microparticles release and bubble formation after an open-sea dive. Appl. Physiol. Nutr. Metab..

[11] Madden D., Thom S.R., Milovanova T.N., Yang M., Bhopale V.M., Ljubkovic M., Dujic Z. (2014). Exercise before SCUBA diving ameliorates decompression-induced neutrophil activation. Med. Sci. Sports Exerc..

[12] Madden D., Thom S.R., Yang M., Bhopale V.M., Milovanova T.N., Ljubkovic M., Dujic Z. (2014). High intensity cycling before SCUBA diving reduces post-decompression microparticle production and neutrophil activation. Eur. J. Appl. Physiol..

[13] Thom S.R., Yang M., Bhopale V.M., Huang S., Milovanova T.N. (2011). Microparticles initiate decompression-induced neutrophil activation and subsequent vascular injuries. J. Appl. Physiol..

[14] Thom S.R., Bennett M., Banham N.D., Chin W., Blake D.F., Rosen A., Pollock N.W., Madden D., Barak O., Marroni A. (2015). Association of microparticles and neutrophil activation with decompression sickness. J. Appl. Physiol..

[15] Thom S.R., Bhopale V.M., Yu K., Yang M. (2018). Provocative decompression causes diffuse vascular injury in mice mediated by microparticles containing interleukin-1beta. J. Appl. Physiol..

[16] Thom S.R., Yang M., Bhopale V.M., Milovanova T.N., Bogush M., Buerk D.G. (2013). Intra-microparticle nitrogen dioxide is a bubble nucleation site leading to decompression-induced neutrophil activation and vascular injury. J. Appl. Physiol..

[17] Yang M., Milovanova T.N., Bogush M., Uzan G., Bhopale V.M., Thom S.R. (2012). Microparticle enlargement and altered surface proteins after air decompression are associated with inflammatory vascular injuries. J. Appl. Physiol..

[18] Yang M., Kosterin P., Salzberg B.M., Milovanova T.N., Bhopale V.M., Thom S.R. (2013). Microparticles generated by decompression stress cause central nervous system injury manifested as neurohypophiseal terminal action potential broadening. J. Appl. Physiol..

[19] Mause S.F., Weber C. (2010). Microparticles: Protagonists of a novel communication network for intercellular information exchange. Circ. Res..

[20] Cabral J., Ryan A.E., Griffin M.D., Riter T. (2018). Extracellular vesicles as modulators of wound healing. Adv. Drug. Deliv. Rev..

[21] Slater T.W., Finkilesztein A., Mascarenhas L.A., Mehl L.C., Butin-Israeli V., Sumagin R. (2017). Neutrophil microparticles deliver active myeloperoxidase to injured mucosa to inhibit epithelial wound healing. J. Immunol..

[22] Thom S.R., Bhopale V.M., Yang M. (2014). Neutrophils generate microparticles during exposure to inert gases due to cytoskeletal oxidative stress. J. Biol. Chem..

[23] Bhopale V.M., Ruhela D., Brett K.D., Nugent N.Z., Fraser N.K., Levinson S.L., DiNubile M.J., Thom S.R. (2021). Plasma gelsolin modulates the production and fate of IL-1β-containing microparticles following high-pressure exposure and decompression. J. Appl. Physiol..

[24] Thom S.R., Bhopale V.M., Arya A.K., Ruhela D., Bhat A.R., Mitra N., Hoffstad O., Malay D.S., Mirza Z.K., Lantis J.C. (2023). Blood-borne microparticles are an inflammatory stimulus in type-2 diabetes mellitus. ImmunoHorizons.

[25] Thom S.R., Bhopale V.M., Yu K., Huang W., Kane M.A., Margolis D.J. (2017). Neutrophil microparticle production and inflammasome activation by hyperglycemia due to cytoskeletal instability. J. Biol. Chem..

[26] Thom S.R., Bhopale V.M., Yang M. (2019). Microparticle-induced vascular injury in mice following decompression is inhibited by hyperbaric oxygen: Effects on microparticles and interleukin-1beta. J. Appl. Physiol..

[27] Thom S.R., Bhopale V.M., Bhat A.R., Arya A.K., Ruhela D., Qiao G., Li X., Tang S., Xu S. (2023). Neuroinflammation with increased glymphatic flow in a murine model of decompression sickness. J. Neurophysiol..

[28] Pospichalova V., Svoboda J., Dave Z., Kotrbova A., Kaiser K., Klemova D., Ilkovics L., Hampl A., Crha I., Jandakova E. (2015). Simplified protocol for flow cytometry analysis of fluorescently labeled exosomes and microvesicles using dedicated flow cytometer. J. Extracell. Vesicles.

[29] Harris F.M., Best K.B., Bell J.D. (2002). Use of laurdan fluorescence intensity and polarization to distinguish between changes in membrane fluidity and phospholipid order. Biochem. Biophys. Acta.

[30] Cypryk W., Nyman T.A., Matikainen S. (2018). From inflammasome to exosome-does extracellular vesicle secretion constitute an inflammasome-dependent immune response?. Front. Immunol..

[31] Gambim M.H., do Carmo A.O., Marti L., Verrissimo-Filho S., Lopes L.R., Janiszewski M. (2007). Platelet-derived exosomes induce endothelial cell apoptosis through peroxynitrite generation: Experimental evidence for a novel mechanism of septic vascular dysfunction. Crit. Care.

[32] Webber R.J., Sweet R.M., Webber D.S. (2019). Inducible nitric oxide synthase in circulating microvesicles: Discovery, evolution, and evidence as a novel biomarker and the probable causative agent for sepsis. J. Appl. Lab. Med..

[33] Noonin C., Thongboonkerd V. (2021). Exosome-inflammasome crosstalk and their roles in inflammatory responses. Theranostics.

[34] Sanchex S.S., Tricerri M.A., Gratton E. (2012). Laurdan generalized polarization fluctuations measures membrane packing micro-heterogeneity in vivo. PNAS.

[35] Khatri N., Sagar A., Peddada N., Choudhary V., Singh Chopra B., Garg V. (2014). Ashish, Plasma gelsolin levels decrease in diabetic state and increase upon treatment with F-actin depolymerizing versions of gelsolin. J. Diab. Res..

[36] Lu C.-H., Lin S.-T., Chou H.-C., Lee Y.-R., Chan H.-L. (2013). Proteomic analysis of retinopathy-related plasma biomarkers in diabetic patients. Arch. Biochem. Biophys..

[37] Lee P.S., Patel S.R., Christiani D.C., Bajwa E., Stossel T.P., Waxman A.B. (2008). Plasma gelsolin depletion and circulating actin in sepsis: A pilot study. PLoS ONE.

[38] Lee P.S., Sampath K., Karumanchi S.A., Tamez H., Bhan I., Isakova T., Gutierrez O.M., Wolf M., Chang Y., Stossel T.P. (2009). Plasma gelsolin and circulating actin correlate with hemodialysis mortality. Am. Soc. Nephrol..

[39] Osborn T.M., Verdrengh M., Stossel T.P., Tarkowski A., Bokarewa M. (2008). Decreased levels of the gelsolin plasma isoform in patients with rheumatoid arthritis. Arthritis Res. Ther..

[40] Peddada N., Sagar A., Ashish, Garg R. (2012). Plasma gelsolin: A general prognostic marker of health. Med. Hypotheses..

[41] Overmyer K.A., Shishkova E., Miller I.J., Balnis J., Bernstein M.N., Peters-Clarke T.M., Meyer J.G., Quan Q., Muehlbauer L.K., Trujillo E.A. (2020). Large-scale multi-omic analysis of COVID-19 severity. medRxiv.

[42] Piktel E., Levental I., Durnás B., Janmey P., Bucki R. (2018). Plasma gelsolin: Indicator of inflammation and its potential as a diagnostic tool and therapeutic target. Int. J. Mol. Sci..

[43] Balestra C., Arya A.K., Leveque C., Virgili F., Germonpre P., Lambrechts K., Lafere P., Thom S.R. (2022). Varying oxygen partial pressure elicits blood-borne microparticles expressing different cell-specific proteins—Toward a targeted use of oxygen?. Int. J. Mol. Sci..

[44] Tung J.W., Parks D.R., Moore W.A., Herzenberg L.A., Herzenberg L.A. (2004). New approaches to fluorescence compensation and visualization of FACS data. Clin. Immunol..

[45] World Medical A. (2013). World Medical Association Declaration of Helsinki: Ethical principles for medical research involving human subjects. JAMA.

